# Review of Applications of Zeolites in Dermatology: Molecular Perspectives and Translational Potentials

**DOI:** 10.3390/ijms26146821

**Published:** 2025-07-16

**Authors:** James Curtis Dring, Matthew Kaczynski, Rina Maria Zureikat, Michael Kaczynski, Alicja Forma, Jacek Baj

**Affiliations:** 1 Department of Forensic Medicine, Medical University of Lublin, ul. Jaczewskiego 8b, 20-090 Lublin, Poland; mattrkaczynski@gmail.com; 2 Department of Correct, Clinical and Imaging Anatomy, Medical University of Lublin, ul. Jaczewskiego 4, 20-090 Lublin, Poland; rmzureikat@outlook.com (R.M.Z.); kaczynskilublin@gmail.com (M.K.); jacek.baj@umlub.pl (J.B.)

**Keywords:** zeolite, clinoptilolite, antiacne, cosmetology, antimicrobial, infection, melanoma, dermatology, antiageing, wound healing

## Abstract

Zeolites, microporous aluminosilicates with tuneable physicochemical properties, have garnered increasing attention in dermatology due to their antimicrobial, detoxifying, and drug delivery capabilities. This review evaluates the structural characteristics, therapeutic mechanisms, and clinical applications of zeolites—including clinoptilolite, ZSM-5, ZIF-8, and silver/zinc-functionalized forms—across skin infections, wound healing, acne management, and cosmetic dermatology. Zeolites demonstrated broad-spectrum antibacterial and antifungal efficacy, enhanced antioxidant activity, and biocompatible drug delivery in various dermatological models. Formulations such as silver–sulfadiazine–zeolite composites, Zn–clinoptilolite for acne, and zeolite-integrated microneedles offer innovative avenues for targeted therapy. Zeolite-based systems represent a promising shift toward multifunctional, localized dermatologic treatments. However, further research into long-term safety, formulation optimization, and clinical validation is essential to transition these materials into mainstream therapeutic use.

## 1. Introduction

Zeolites are crystalline aluminosilicates renowned for their porous architecture and ion exchange capabilities, which have led to their diverse applications in environmental and industrial sectors. In recent years, growing attention has been directed toward their biomedical potential, particularly in dermatology. Their ability to adsorb toxins, modulate local inflammation, and act as carriers for antimicrobial or therapeutic agents makes them attractive candidates for managing various skin conditions [1,2,3,4,5,6]. These include acne, atopic dermatitis, chronic wounds, and fungal infections—areas where conventional therapies often fall short due to antimicrobial resistance or poor skin tolerance.

Clinoptilolite, ZSM-5, ZIF-8, and metal-doped variants such as silver- and zinc-functionalized zeolites exhibit promising anti-inflammatory, antimicrobial, and detoxifying effects when applied topically. Advancements in nanotechnology have further expanded their application in hydrogel dressings, microneedles, and composite wound care systems. This review offers a comprehensive evaluation of zeolite structures, their mechanisms of action in dermatological applications, clinical efficacy, and future therapeutic potential. Particular attention is given to the limitations of conventional treatments, including antimicrobial resistance, skin irritation, systemic medication, and formulation challenges, and how zeolite-based strategies may address these issues. By integrating decades of molecular and clinical research, this review highlights the translational relevance of zeolites and aims to bridge the gap between laboratory findings and evidence-based dermatological care.

## 2. Zeolites and Skin Care Formulation

There has been an increasing demand for effective and biocompatible detoxification agents, particularly within medical and dermatologic applications. Zeolites have attracted significant attention due to their remarkable adsorptive properties and ability to remove a wide range of environmental toxins [1,2,3,6,7,8,9]. Their highly porous structure and ion exchange capabilities make zeolites ideal for capturing pollutants, heavy metals, and volatile organic compounds (VOCs), contributing to enhanced dermatological health and systemic detoxification [1,2,3,6,7,10,11].

In addition to their established use in industrial and environmental sectors, zeolites have recently emerged as promising medical and cosmetic dermatology agents. Their non-toxic and non-irritating nature makes them suitable for direct skin contact, offering multiple benefits such as pollutant adsorption, drug delivery, sebum regulation, and gentle exfoliation [2,3,6,11,12,13,14]. Zeolites benefit skin care by removing environmental pollutant accumulation, which can lead to dermatological diseases. Nanotechnology and material sciences advancements have refined their functionality, improving their ability to bind and eliminate harmful substances from the skin’s surface. Recent studies have shown that modified zeolite formulations can effectively adsorb particulate matter (PM2.5 and PM10), heavy metals, and other urban pollutants, thereby reducing oxidative stress and minimizing the risk of inflammatory skin conditions like atopic dermatitis and acne [1,3,12,14,15].

Due to their unique adsorption properties, zeolites are applied in several biomedical fields. Firstly, their capacity for detoxification stems from their high surface area and ion exchange capacity, enabling the sequestration of heavy metals such as lead (Pb), cadmium (Cd), and nickel (Ni) from the skin’s surface [1,2,14]. While much of the foundational knowledge surrounding detoxification derives from environmental and gastrointestinal studies, recent in vitro research—including dermal formulations tested with metal-spiked creams—has demonstrated zeolites’ ability to remove toxic ions directly from the skin via preliminary studies [3,14]. In drug delivery, zeolites’ adjustable pore sizes and adsorption profiles allow for the controlled release of therapeutic agents, including erythromycin and zinc, offering improved stability and reduced systemic exposure [2,14,16]. They also contribute to moisture regulation, maintaining stability in topical formulations through controlled hydration and desiccation properties [2,14].

Furthermore, zeolites can support catalytic activity by acting as carriers for enzymes that mimic oxidative stress-regulating functions. Though zeolites themselves do not exhibit enzymatic activity, they can stabilize and immobilize enzymes like peroxidase and superoxide dismutase through surface adsorption or covalent bonding, thereby enhancing enzyme durability and mimicking natural antioxidant defence mechanisms [2,3,17]. In cosmetic and therapeutic skin care, zeolites aid in the adsorption of toxins, microbial contaminants, and excess sebum while facilitating the delivery of active compounds to target sites, as evidenced in acne management and anti-inflammatory applications [2,12,14,15,18]. Collectively, these attributes position zeolites as adaptable components in contemporary dermatological formulations, connecting eco-friendly detoxifying capabilities with clinically focused skin treatments.

This subsection examines the role of zeolites in medical detoxification, highlighting their adsorptive mechanisms, potential for systemic toxin removal, and applications in dermatology. By analyzing recent studies and technological advancements, we emphasize the integration of zeolites into clinical and environmental skincare solutions, bridging sustainable materials with medical innovations.

### 2.1. Structure and Adsorption

Zeolites are a class of minerals characterized by a highly porous, three-dimensional lattice structure composed of aluminum oxide (Al_2_O_3_) and silicon dioxide (SiO_2_), belonging to the tectosilicate mineral group [1,2,3,19,20]. Their unique framework, consisting of corner-sharing TO_4_ tetrahedra (where T represents silicon or aluminum atoms), enables precise molecular separations at a sub-nanometre scale [2,20,21]. Since their commercial introduction in the 1950s, zeolites have proven to be invaluable in various industries due to their tuneable composition, high stability, and selective adsorption properties. Zeolites are widely utilized in environmental, industrial, and medical applications. 

**Figure 1:**
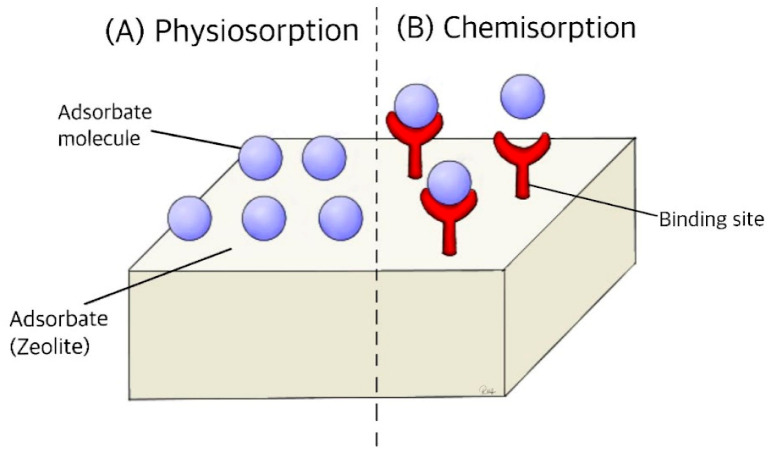
Zeolites function via two primary adsorption mechanisms: (**A**) Physisorption: Weak intermolecular forces allowing reversible adsorption, useful in drug delivery [2]. The adsorbate molecule, such as a drug, weakly binds to the surface of the zeolite to allow for a reversible reaction. (**B**) Chemisorption: Stronger interactions enable selective toxin removal and biosensor applications [2]. Covalent or ionic bonds form on the surface of the zeolite through a binding site.

The empirical formula of zeolites, reflects the Si/Al ratio, which influences key properties such as polarity, adsorption capacity, and thermal stability (Figure 1) [2,9,14]. The charge imbalance from aluminum incorporation is balanced by extra-framework cations, including sodium (Na^+^), calcium (Ca^2+^), and potassium (K^+^), contributing to the material’s ion exchange capacity [2,7,9,19,20,22,23,24]. This ion exchange ability is particularly valuable for removing toxic cations such as lead (Pb^2+^), mercury (Hg^2+^), and cadmium (Cd^2+^) [2,7,9,12,19,23,25].

Mz + y[Si_1−x_Al_x_O_x_]_x−_

By substituting Si and Al with elements such as boron (B), germanium (Ge), cobalt (Co), iron (Fe), and titanium (Ti), different zeolite variants with unique adsorption and catalytic properties are created [2,3,14]. Notable variants include aluminophosphates (AlPOs) and silicoaluminophosphates (SAPOs), which affect stability and selectivity in adsorption and catalysis (Table 1) [2].

**Table 1:** Zeolite Classification and Properties.

Category	Type	Properties	Ref.
Si/Al Ratio	Low-silica zeolites (Si/Al < 2)	High polarity, strong adsorption of polar molecules like water.	[2,14,26,27]
	Medium-silica zeolites (Si/Al = 2–5)	Intermediate polarity.	[2,14,26,27]
	High-silica zeolites (Si/Al > 5)	Lower polarity, higher thermal/chemical stability, often hydrophobic.	[2,14,26,27]
	Pure-silica zeolites (Zeosils)	No aluminum, highly stable and hydrophobic.	[2,14,26,27]
Porosity	Cages	Small enclosed voids for molecular sieving.	[2,20,21,24,28]
	Cavities	Larger polyhedral voids facilitating diffusion.	[2,20,21,24,28,29]
	Channels	Extended pore systems allowing molecular transport.	[2,20,21,24,29]
Pore Size	Small pores (3–5 Å, 8–9 TO_4_ rings)	Suitable for water and small molecules.	[2,16,24,27]
	Medium pores (5–6 Å, 10-ring)	Balances selectivity and transport.	[2,16,24,27]
	Large pores (6–7.5 Å, 12-ring)	Increased molecular accessibility.	[2,16,27]
	Extra-large pores (>7.5 Å, >12-rings)	Allow larger molecules to pass through.	[2,16,27]
Diffusion Types	Micropore diffusion	Predictable movement within the unit cell.	[2,30]
	Mesopore diffusion	Influenced by adsorbent–adsorbate interactions.	[2,30]
	Macropore diffusion	Dominated by bulk-phase molecular interaction.	[2,30]
Biomedical Applications	Detoxification	Removal of heavy metals and toxins from biological systems.	[2,3,14,30]
	Controlled drug delivery	Slow-release medications, such as erythromycin and zinc in topical acne treatments.	[2,12,13,14,16,18]
	Selective adsorption of biomolecules	Enabling target therapeutic applications.	[2]
Synthesis Methods	Hydrothermal synthesis	Mimics natural crystallization using oxidized silicon and aluminum sources, allowing precise drug interactions and release.	[2,9,14,18]
	Novel synthesis techniques	Includes interzeolite conversions, topotactic transformations, ionothermal synthesis, and microwave-assisted synthesis to enhance properties.	[2]
Post-Synthetic Modifications	Ion exchange	Incorporating therapeutic metals (e.g., silver, copper) to enhance antimicrobial activity.	[2,9,12,23,24,31,32]
	Surface functionalization	Modifying the zeolite surface to improve hydrophobicity and selectivity.	[2,7,13,14,22]
	Calcination and steaming	Increasing mesoporosity and adsorption efficiency through controlled thermal treatments.	[2,13,14]
	Shaping and structuring	Granulating zeolites into beads or pellets for improved handling in topical and industrial applications.	[2,7,9,14]

### 2.2. Heavy Metal Adsorption

A study conducted on zeolite creams containing varying concentrations (1% and 3%) showed that zeolites possess significant adsorption capacity for heavy metals such as cadmium (Cd), lead (Pb), chromium (Cr), nickel (Ni), and cobalt (Co) [3]. The adsorption of Cd, Ni, and other metals was significantly higher in the 3% zeolite cream formulation compared to the placebo, suggesting that zeolites can effectively remove heavy metals from the skin, especially in higher concentrations. Statistical analysis confirmed enhanced adsorption of Cd and Ni at 3% (*p* < 0.05) [3].

In this study, the preparation involved two zeolite creams, spiked with metal solutions and subjected to acid digestion followed by metal quantification using ICP-OES. The results from these formulations were analyzed to determine the extent of heavy metal retention, showing that zeolites act as potent cation exchangers, effectively binding toxic metals through their microporous crystalline structure [3]. The ability to adsorb heavy metals on the skin is vital for preventing their absorption into the body, protecting skin health, and reducing environmental exposure, thereby mitigating the potential health risks associated with these toxic elements and systemic toxicity [2,3,7,12,25]. Heavy metals such as Pb, cadmium (Cd), and mercury can penetrate the skin barrier, disrupt cellular function, and trigger free radical formation, accelerating skin damage.

### 2.3. Clinoptilolite

Zeolite clinoptilolite (ZC) is one of the most abundant naturally occurring zeolites, widely utilized in medical, environmental, and industrial applications [7,22]. Due to its stability and ion exchange properties, ZC has gained attention in research for gastrointestinal health, detoxification, and immune modulation.

Clinoptilolite’s high Si/Al ratio provides enhanced structural stability, allowing it to withstand acidic conditions such as those in the gastrointestinal tract (GIT) [10,14,22]. Unlike low-silica zeolites like Zeolite A, which easily degrade in acidic environments, clinoptilolite maintains its integrity under prolonged exposure to high temperatures (up to 750 °C for 12 h) and remains stable in biological systems [10,22]. These properties make it an effective material for medical applications, particularly detoxification and antioxidant activity. Additionally, the material’s high affinity for cations (e.g., Na^+^, K^+^) enables it to regulate electrolyte balance and facilitate cation uptake in ion exchange settings with substances such as zinc (Zn^2+^) [10,12,22].

#### 2.3.1. Antioxidant and Detoxification Properties

##### Antioxidant Effects

Reactive oxygen species (ROS) such as peroxides, superoxides, and hydroxyl radicals play a dual role in cellular homeostasis and disease pathology. While moderate ROS levels are necessary for cellular functions, excessive ROS can cause oxidative stress, leading to DNA damage, protein oxidation, and lipid peroxidation [10,17,22,25]. Clinoptilolite contributes to antioxidant defence by:

- Restoring Metal Homeostasis: Through ion exchange, clinoptilolite releases essential cations such as calcium (Ca), manganese (Mn), zinc (Zn), and magnesium (Mg), which are required for the activation of antioxidant enzymes [7,9,12,14,22,25].
- Reducing Lipid Oxidation: By lowering LDL cholesterol levels, clinoptilolite indirectly enhances antioxidant effects, reducing oxidative stress [25].
- Modulating Hydrogen Peroxide Levels: Tumour cells exhibit elevated hydrogen peroxide (H_2_O_2_) levels, which regulate key signalling pathways and modulate oxidative stress. Clinoptilolite may interact with H_2_O_2_, breaking it down into hydroxyl or hydroperoxyl radicals, potentially influencing tumour cell viability [25].

**Figure 2:**
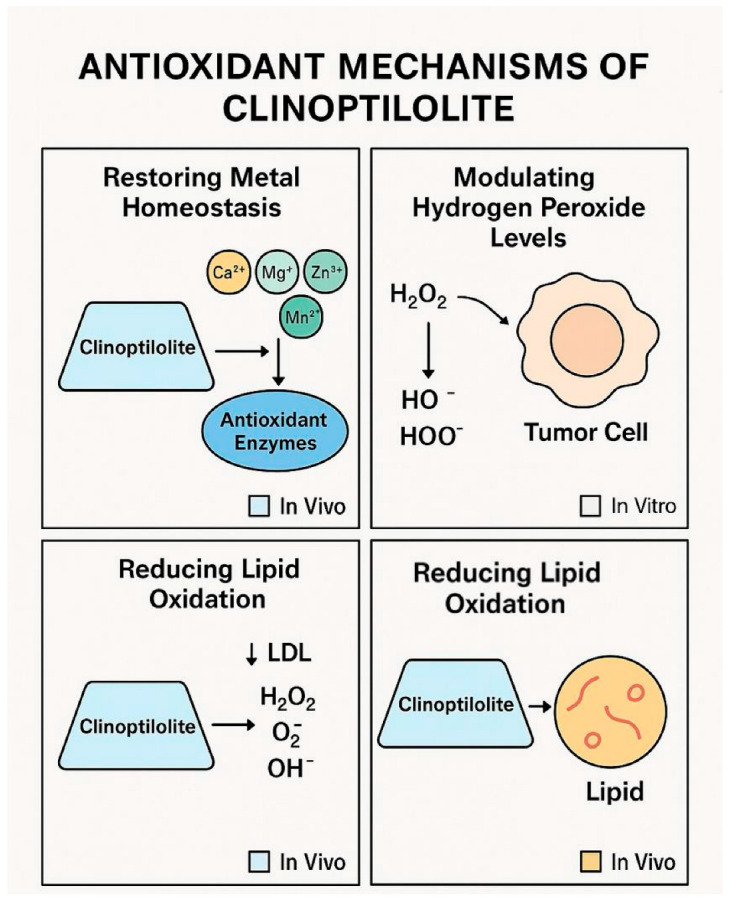
Summary of antioxidant mechanisms of clinoptilolite relevant to dermatological and systemic applications.

Clinoptilolite contributes to oxidative balance by restoring trace metal homeostasis essential for antioxidant enzyme function, modulating hydrogen peroxide levels, and reducing lipid peroxidation. Evidence for these actions is primarily derived from in vitro and preclinical studies [7,9,12,14,22,25], as shown in Figure 2.

##### Immunomodulatory Effects

Clinoptilolite has demonstrated the ability to modulate immune responses, particularly in the gut and on the skin. It has been observed to:

- Reduce Antibiotic-Resistant Bacteria: Long-term supplementation with clinoptilolite has been associated with a decreased prevalence of antibiotic-resistant *Escherichia coli* strains and *Propionibacterium acnes* [9,18,25].
- Stimulate Immune Cell Activity: Clinoptilolite supplementation increases the activation of B lymphocytes (CD19+), T helper cells (CD4+), and activated T-lymphocytes (HLA-DR+), while reducing natural killer (NK) cell (CD56+) counts [25].

##### Potential Antitumor Effects

Preliminary in vitro studies suggest that clinoptilolite may exert antitumor effects. Tumour cells often exhibit high H_2_O_2_ levels, which can modify cysteine residues on antioxidative enzymes, leading to their deactivation. Clinoptilolite’s interaction with H_2_O_2_ may induce oxidative stress in tumour cells, a mechanism similar to that observed with silica particles. However, further research is needed to validate these findings in clinical settings [14,25].

#### 2.3.2. Combination with Zinc: Enhanced Medical Benefits

Clinoptilolite has shown significant promise as a carrier for Zn^2^⁺ ions, particularly in dermatological and medical applications [12,14,33]. When combined with zinc, clinoptilolite serves as a controlled-release system, ensuring prolonged zinc bioavailability in the skin and other biological environments [12,14]. This combination offers several benefits [12]:

- Antimicrobial Properties: Zinc has well-documented antimicrobial activity against *Propionibacterium acnes* and *Staphylococcus aureus*, to name a few, making clinoptilolite-Zn^2^⁺ formulations effective in treating acne and other bacterial skin conditions [12,15].
- Enhanced Wound Healing: Zinc plays a crucial role in tissue regeneration, and its controlled release via clinoptilolite enhances wound healing processes while minimizing cytotoxic effects [7,12,15,33].
- Sebum Regulation: Clinoptilolite-Zn^2^⁺ complexes contribute to the regulation of sebum production, making them particularly beneficial for individuals with oily or acne-prone skin [12,31].
- Anti-Inflammatory Effects: Zinc’s anti-inflammatory properties are complemented by clinoptilolite’s ability to neutralize harmful free radicals, reducing skin irritation and redness [12,22].

#### 2.3.3. Evaluation of Clinoptilolite-Rich LacVen Rock as a Carrier

Clinoptilolite-rich LacVen rock has been identified as a cost-effective and readily available alternative to pure zeolites, making it a practical choice for developing topical treatments. The reproducibility of this material ensures consistent experimental results, essential in pharmaceutical applications.

Several key properties of the LacVen rock contribute to its suitability as a medical carrier:

- Fast zinc release: Rapid ion exchange in physiological and buffer solutions guarantees immediate bioavailability upon topical application [12]
- Erythromycin loading capacity: LacVen was measured to have an erythromycin loading of 28.9%, with 85% of the drug contacting the carrier successfully loaded onto it [12]
- Safety profile: Low levels of potentially harmful elements (As, Cd, Cr, Cu, and Pb) make it suitable for medical use [12]
- Pharmaceutical compliance: The whitish colour of the powder improves patient compliance for topical treatments [12]

### 2.4. Dermatologic and Cosmetic Formulation

Recent studies have explored the use of micronized natural zeolitic volcanic tuff (MZ) in cosmetic formulations, particularly exfoliating masks [7]. Micronization enhances the surface area and cation exchange capacity of zeolites, improving their functional properties without altering their structural integrity. Two formulations of 5% MZ, with particle sizes of 100–125 μm and 125–250 μm, were evaluated for cosmetic suitability. X-ray fluorescence (XRF), X-ray diffraction (XRD), and Fourier transform infrared (FT-IR) spectroscopy confirmed MZ’s composition and structure. Physicochemical assessments, including pH, cation exchange capacity (CEC), and apparent density, showed no significant changes post micronization [7].

Cosmetic formulations containing MZ exhibited a stable pH of 4.3, a pleasant texture, and good spreadability without colour alteration. Stability testing over 15, 30, and 60 days under various temperature conditions confirmed high formulation stability, supporting MZ as a promising ingredient for exfoliation and detoxification [7].

#### 2.4.1. UV Protection and Sunscreens

A growing concern in dermatology is the generation of ROS by titanium dioxide (TiO_2_), a common sunscreen ingredient, upon UVA exposure. To mitigate this, researchers have encapsulated TiO_2_ within NaY zeolites. Shen et al. (2006) demonstrated that this encapsulation significantly reduces UVA-induced ROS generation in human fibroblasts and yeast cells, potentially enhancing sunscreen safety [3,17].

NaY zeolite alone reduced ROS levels, and encapsulated TiO_2_ further amplified this reduction, effectively doubling the decrease in oxidative stress compared to zeolite alone. These findings highlight zeolite encapsulation as a viable strategy for improving sunscreen efficacy and reducing cellular damage [2,3,17,34].

As mentioned, encapsulation with zeolites has been shown to improve the safety of organic sunscreens, particularly oxybenzone (OXB), which is associated with photoallergy and phototoxicity [2,21]. Encapsulating OXB within NaY zeolites forms a supramolecular sunscreen system that retains UV protection while reducing direct skin contact, thereby minimizing allergic reactions and toxicity.

Studies confirm that zeolite-encapsulated OXB maintains its UV absorption properties and enhances photostability, reducing environmental degradation (e.g., TiO_2_-induced degradation was lowered from 60% to 20%) [34]. In vitro studies with human epidermal keratinocytes (HEKs) demonstrated improved cell viability and reduced DNA damage after UV exposure, as evidenced by alkaline comet assay analysis. Furthermore, the broadening of the UVA absorption spectrum enhances protective efficacy while reducing adverse interactions between sunscreen ingredients [17,34].

However, formulation challenges remain. Zeolite particles, due to their size, can cause opacity in sunscreen products, affecting esthetic appeal and application ease. However, physical sunscreen options could offer this narrative as a protective feature. To address the esthetic drawbacks of zeolite opacity, recent studies have focused on modifying particle characteristics to improve visible transparency. Particle size plays a critical role in light scattering; zeolites larger than 50 μm tend to produce a whitening effect on the skin surface, compromising cosmetic performance [35]. Fantini demonstrated that reducing the average particle size of LTL-type zeolites to below 20 μm through wet grinding significantly diminished opacity, resulting in a smoother texture, better dispersion in emulsions, and improved visual transparency. In parallel, surface modification strategies such as encapsulating organic UV filters—like avobenzone and octinoxate—within zeolite frameworks (forming ZEOfilters) have been shown to further enhance photostability while minimizing visible residues [36,37]. These adjustments not only improve the sensory profile of sunscreen formulations but also reduce the amount of active filter required, lowering the risk of photodegradation and irritation. While promising, further research is needed to optimize coating techniques, assess long-term stability under real-use conditions, and evaluate the regulatory implications of using micronized or surface-modified particles in commercial products.

#### 2.4.2. Anthocyanidin–Zeolite Complexes for Skin Protection

Another promising advancement in zeolite-based dermatologic formulations involves anthocyanidin–zeolite (A–Z) complexes, which have shown potential in improving cell viability while reducing toxicity in skin cells. A study by Aroonsri et al. (2013) investigated the effects of A–Z complexes on human epidermoid carcinoma keratinocytes (A431) and human forehead fibroblasts (HFFs) using real-time cell analysis [38].

The study utilized a mesoporous zeolite with 5 nm pores, which provided an optimal structure for encapsulating anthocyanidin extracted from Clitoria ternatea Linn flowers, with delphinidin as the primary bioactive component [38]. The anthocyanidin was absorbed into the zeolite to form the A–Z complex, and its cytotoxicity was evaluated against individual anthocyanidin and zeolite treatments [38].

The findings indicated that the A–Z complex significantly improved cell survival while demonstrating lower IC50 values compared to either anthocyanidin or zeolite alone. This suggests that the combination reduces cytotoxic effects, particularly in A431 and HFF cells, where immediate drops in cell viability were less pronounced upon exposure [38].

Anthocyanidins are well-documented antioxidants with protective effects against environmental stressors, such as UV radiation and pollution [3,34,38]. Their antiageing properties stem from their ability to:

- Reduce oxidative stress and protect cells from free radical damage.
- Enhance skin regeneration and healing.
- Provide soothing and hydrating effects, making them suitable for irritated skin formulations.

The A-Z complex offers a dual benefit by mitigating toxicity while improving cell survival, positioning it as a promising ingredient for dermatologic and cosmetic applications aimed at skin protection and hydration [38].

### 2.5. Zeolites in Odour Control

Body odour results from the bacterial decomposition of sweat components, primarily by *Staphylococcus epidermidis*, *Micrococcus* spp., and *Corynebacterium* spp. Conventional deodorants employ various strategies such as perspiration suppression (e.g., chlorohydroxyaluminum), bacterial reduction (e.g., triclosan or benzalkonium chloride), and deodorization (e.g., zinc oxide, MgO-SiO_2_ complex, and flavonoids) [15]. Safety concerns in antimicrobial agents such as triclosan have necessitated the need for safer alternatives. 

Silver-exchanged zeolite (Ag–zeolite) offers a safe, long-lasting antibacterial effect [2,3,9,31,32], comparable to triclosan, but without the associated risks [31]. A single application provides up to 24 h of odour prevention, with a 10% Ag–zeolite spray outperforming 0.2% triclosan in bacterial reduction and deodorization [31].

## 3. Uses of Zeolites in Wound Healing and Infection Management

Zeolite-based materials present a paradigm shift in wound healing and infection control, especially with the advent of zeolitic imidazolate framework-8 (ZIF-8), which demonstrates exceptional biocompatibility, drug loading capabilities, and antimicrobial action [39]. The exploration of zeolites extends to various wound scenarios, encompassing peri-implantitis, diabetic ulcers, the creation of artificial neutrophils, oral mucosal ulcers, and general cutaneous wound repair, providing a broad spectrum of application [40]. The core focus revolves around the ability of ZIF-8 to modulate inflammatory responses, encourage the regeneration of tissues, and lower the occurrence of infections, marking it as a pivotal component in advanced wound care strategies. Clinoptilolite, a naturally occurring zeolite, has also shown promise in the realm of cutaneous wound healing, indicating the diverse applicability of zeolite minerals in dermatological treatments. Although zeolites have shown efficacy in hastening wound recovery and minimizing the need for antibiotics, further extensive clinical research is essential to refine their formulations and fully understand their long-term safety profiles [41]. The forthcoming sections will thoroughly analyze recent studies focusing on the use of zeolites in wound treatment, underscoring their relevance in addressing peri-implantitis, diabetic ulcers, artificial neutrophil construction, microneedle therapies for oral ulcers, and the creation of clinoptilolite-based wound dressings.

### 3.1. Wound Healing Applications

Wound healing is a complex biological process involving inflammation, tissue proliferation, and remodelling [42]. Impaired healing, commonly seen in diabetic ulcers, peri-implantitis, and chronic wounds, necessitates novel therapeutic approaches [43]. Zeolite-based materials, particularly zeolitic imidazolate framework-8 (ZIF-8), have gained significant attention due to their high porosity, biocompatibility, and multifunctionality [44].

ZIF-8 is a metal–organic framework (MOF) composed of zinc ions and 2-methylimidazolium salts, offering high drug loading capacity, controlled release mechanisms, and antimicrobial properties [42]. Although long-term data on ZIF-8 use remain limited, the trials and experimental studies reviewed here report a favourable safety profile and effective short-term outcomes.

#### 3.1.1. Zeolitic Imidazolate Framework-8 (ZIF-8) in Wound Healing

##### ZIF-8 in Peri-Implantitis Treatment

Peri-implantitis is a serious inflammatory condition affecting dental implants, characterized by soft and hard tissue destruction due to bacterial infection. Hu et al. (2024) developed a hydrogel composite (HMME@ZIF-8/Met/BMP-2@PLGA/GelMA) incorporating [42]:

- Hematoporphyrin monomethyl ether (HMME)—enhances reactive oxygen species (ROS) production, leading to bacterial cell damage.
- Metformin (Met)—reduces pro-inflammatory cytokines (IL-6, TNF-α), suppressing early-stage inflammation.
- Bone morphogenetic protein-2 (BMP-2)—stimulates bone regeneration.

In vivo studies using *P. gingivalis*-infected rats demonstrated significant inflammation reduction and enhanced healing when treated with the hydrogel, compared to untreated controls. The study confirmed that ROS production disrupted bacterial virulence, while metformin further inhibited inflammation (Table 2) [42].

**Table 2:** Zeolite Types, Applications, and Experimental Outcomes.

Zeolite Type	Key Application	Experimental Outcome	Study Type	References
Clinoptilolite	Wound healing, detoxification, acne therapy	Reduced inflammation, oxidative stress; enhanced tissue regeneration; safe in humans	In vitro, in vivo, clinical	[12,14,22,25,43,44,45,46,47,48,49,50]
ZIF-8	Drug delivery, antimicrobial, wound healing	Controlled Zn^2^⁺ release, antimicrobial, enhanced healing in diabetic ulcers and oral ulcers	In vitro, in vivo (animal, limited human data)	[36,37,38,39,40,41,42,51,52,53,54,55,56,57,58,59]
ZSM-5	Antibiotic delivery, antibiofilm	Sustained antibiotic release, reduced biofilm formation, promoted tissue regeneration	In vitro, animal models	[60,61,62,63,64,65,66,67,68,69,70]
Zeolite X	Broad-spectrum antimicrobial	Effective against *E. coli*, *S. aureus*; enhanced Ag⁺ release improves potency	In vitro (antimicrobial assays)	[71,72]
NaY Zeolite	Burn wound antimicrobial composite	Controlled AgSD release; prolonged antimicrobial activity; enhanced wound healing	In vitro, formulation stability tests	[73,74,75,76,77,78]
Ag-Zeolite	Deodorant, long-lasting antibacterial	Outperformed triclosan, provided 24 h odour protection	In vitro, clinical comparison with triclosan	[2,3,9,31,32]
CaCu-Zeolite Gauze	Haemostasis and infection control	Faster clotting, antibacterial action; biocompatible and safe	In vitro, in vivo (animal studies)	[79,80,81,82]
Detoxsan^®^ (Clinoptilolite + Mordenite)	Fungal infections, intertrigo	Improved skin recovery, no adverse effects	Clinical observational study	[15,83,84]
Gold-Zeolite Nanocomposites	Antiageing and skin brightening	Enhanced wrinkle reduction and melanin reduction (clinical trial)	Human clinical trial	[13,85,86]

##### ZIF-8 and Diabetic Ulcers

Diabetic ulcers result from poor vascularization, immune dysfunction, and prolonged inflammation. He et al. (2024) [43] investigated a nanoenzyme-modified hydrogel (CeO_2_–Y@ZIF-8@Gel), demonstrating:

- Oxidative stress reduction via ROS scavenging and mitochondrial protection.
- Enhanced immune regulation through macrophage polarization (M1 → M2 transition).
- Antimicrobial effects when modified with quaternary ammonium salts (QAS).

Diabetic mice treated with CeO_2_–Y@ZIF-8@Gel exhibited the fastest wound healing time (14 days), along with lower infection markers (CRP, PCT) [43]. These findings suggest that ZIF-8-based therapies could reduce antibiotic dependence while accelerating natural healing processes.

#### 3.1.2. ZIF-8 Encapsulation Method in Infection Control

Neutrophils play a crucial role in immune defence but have a short lifespan (~24 h) and lack proliferative ability. Jiang et al. (2024) [44] developed an artificial neutrophil (AN) system by encapsulating glucose oxidase and chloroperoxidase inside a ZIF-8 framework, allowing for:

- Chemotaxis towards H_2_O_2_ gradients, mimicking natural neutrophil migration.
- HClO synthesis, enhancing antimicrobial efficacy.
- Enzyme stabilization, preventing leakage and maintaining activity under physiological conditions.

In vitro and in vivo studies confirmed that ZIF-8 encapsulation improved AN stability and functionality, paving the way for synthetic immune cell therapies (Table 2) [44].

#### 3.1.3. Zeolite-Based Antimicrobial Therapies

Oral mucosal ulcers are highly susceptible to bacterial infections, delaying healing. Yu et al. (2024) [45] designed a hyaluronic acid/gelatin methacryloyl microneedle (HA/GelMA MN) loaded with ZIF-8, which:

- Stably released zinc ions, damaging bacterial envelopes.
- Exhibited sustained antimicrobial effects without antibiotics.
- Enhanced wound healing and tissue regeneration.

**Figure 3:**
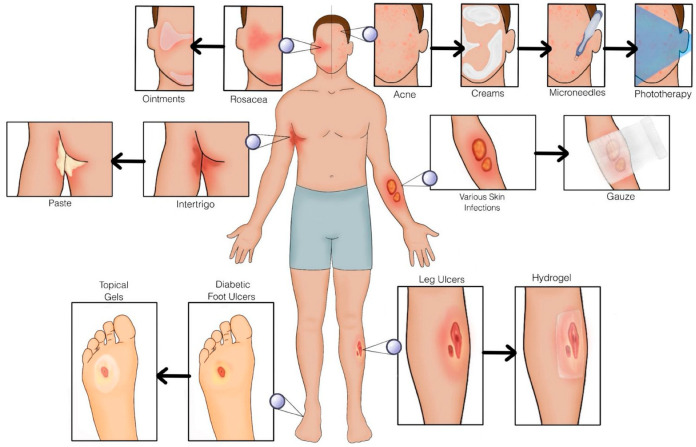
Schematic presentation of zeolite-based topical therapies for common dermatological conditions and infections. Visual representation of therapies discussed in this review for reader reference, each therapy presented is congruently discussed in greater detail.

**Figure 4:**
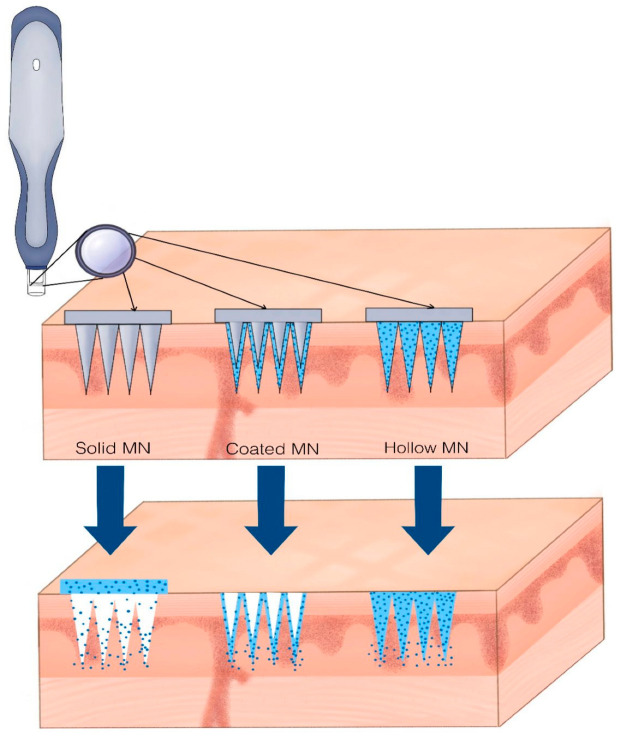
Illustration of solid, coated, and hollow microneedle (MN) systems used in dermatological and cosmetological delivery. Solid MNs create microchannels for enhanced topical absorption, coated MNs deliver actives pre-applied to their surface, and hollow MNs enable direct infusion into the dermis. Zeolite-integrated formulations, such as ZIF-8 and Zn–clinoptilolite, have been incorporated into MNs to improve the delivery of anti-ageing, depigmenting, and antiacne agents through controlled, sustained release while enhancing dermal bioavailability and treatment precision [13,87,88,89,90,91,92,93].

These findings suggest that ZIF-8-based microneedle therapy may serve as a non-invasive treatment for oral wounds [45]. Figure 3 shows microneedles alongside alternative therapeutic modalities using zeolites, whereas Figure 4 elucidates the functionality of microneedles.

#### 3.1.4. Clinoptilolite in Cutaneous Wound Healing

Clinoptilolite is a naturally occurring zeolite with strong adsorption properties, making it suitable for wound dressing applications [46]. A randomized phase I clinical trial assessed topical purified clinoptilolite tuff (PCT) application in 12 participants with 4 mm biopsy wounds. The study found:

- Comparable wound healing rates to standard care (SoC).
- Significant bacterial adsorption, reducing infection risk.
- Minimal erythema and inflammation over 14 days.

While no major adverse effects were observed, further research is needed to explore long-term clinical efficacy and potential dermatological applications [46]. Figure 3 depicts the application of gauze and dressings to the human body.

Deinsberger et al. assessed the topical application of purified clinoptilolite tuff (PCT), a naturally occurring zeolite, in the artificial skin wounds of healthy volunteers in a phase I clinical trial conducted in 2022 [46]. With no indications of delayed wound healing, infection, or local irritation, the trial proved that PCT is safe and well tolerated. PCT-treated wounds showed a different histological profile, with fewer αSMA-positive myofibroblasts and more CD68-positive macrophages, indicating modulation of the contractile and inflammatory phases of repair, even though the wound healing parameters did not differ significantly from the standard of care. Its potential for use in wound care was highlighted when zeolite particles were found in healed tissues without causing any negative effects. More research is necessary in chronic or infected wound models where clinoptilolite’s ability to adsorb exudate, bacterial toxins, and malodorous biogenic amines may provide clear clinical benefits (Table 2) [25,46,47,48,49,50,79,80,81].

#### 3.1.5. Calcium Copper Zeolite Gauze in Cutaneous Wound Healing

Zeolites have shown promising results in uses as gauze for cutaneous wounds when combined with other components, namely calcium and copper [82]. The FDA has approved zeolite–calcium gauzes as early as 2002, according to Guo et al. (2023) [94]. Zeolite and calcium gauzes have also been assessed for their uses in diabetic foot ulcers [95]. With the added benefit of copper’s antimicrobial effects, there is great potential for a zeolite-based gauze for healing infected cutaneous wounds. Wang et al. (2024) performed a study synthesizing, evaluating, and testing calcium copper zeolite dressings, finding [82]:

- The absorption of water in the blood to concentrate coagulation factors and blood cells, which in turn activates the clotting cascade.
- The antibacterial effects of copper through the release of copper ions, which depolarize bacterial cell membranes, promote the production of ROS, have an influence on enzyme activity and DNA damage.
- The whole-blood clotting time of CaCu-ZG gauze sharply decreased from 509 s to 282 s when compared with standard medical gauze, with improved antibacterial activity found both in vivo and in vitro (Table 2).

Wang et al. (2024) presented a dual-function material—CaCu-ZG, a calcium–copper ion-exchanged zeolite gauze—in response to the demand for sophisticated wound dressings with both haemostatic and antibacterial efficacy [82]. The dressing achieved > 99.9% inhibition in vitro and significantly decreased bacterial loads in vivo by utilizing the porous structure of zeolite for controlled ion exchange, which facilitated rapid Ca^2+^-induced coagulation and Cu^2+^-mediated antibacterial effects against *E. coli* and *S. aureus*. After several uses, CaCu-ZG retained its structure and effectiveness better than commercial substitutes and regular gauze. In mice, in vitro testing found it demonstrated outstanding biocompatibility, little haemolysis, no cytotoxicity, and systemic safety. CaCu-ZG promoted quicker closure, epithelial repair, and collagen deposition in a full-thickness infected wound model. No in vivo adverse effects were reported.

Based on the superb procoagulant effects that are not affected by the copper ion exchange, efficiency of antibacterial effect, good biocompatibility, outstanding durability, and stability, there is a promising future in the use of CaCu-ZG for infected cutaneous wound healing. Figure 3 depicts gauze application simply on these wounds. Traditionally wound dressings would need to be monitored and changed [14]. Of course, further investigations warrant their potential use clinically.

#### 3.1.6. Antimicrobial and Wound Healing Properties of NO-Loaded Zeolite Ointment

Impaired endogenous NO production has been associated with impaired wound healing. Neidraur et al. (2014) proposed combining NO with zeolites into an ointment for topical applications to promote faster cutaneous wound healing [80]. This study initiated the introduction of zeolite as an ointment base, slowing the release of NO and allowing for the longer effects of NO’s healing acceleration properties.

In vitro assessment on obese Zucker rats found the ointment was effective against a broad spectrum of common Gram-positive and -negative wound pathogens (e.g., *E. coli*, *A. baumanni*, *S. epidermidis*, and MRSA). Wounds treated with the NO–zeolite ointment showed no signs of inflammation (e.g., erythema or swelling), when compared with the control ointment while having shorter wound closure times. After the assessment of animal subjects, and results finding improved wound healing time, as well as broad-spectrum antibacterial activity, the NO–zeolite ointment showed promise for use in cutaneous wound healing.

Zeolite-based nitric oxide (NO) delivery provides a new approach for regulated topical immunomodulation. In a clinical study, Ze–NO—a manganese-loaded zeolite—was found to release biologically active NO without causing the strong inflammation seen with acidified nitrite formulations [8]. Unlike acidified NO_2_^−^, which caused significant neutrophilic infiltration, ulceration, and Langerhans cell depletion from the generation of hazardous reactive nitrogen and oxygen species [8,96,97,98,99], Ze–NO caused only slight erythema and the moderate dermal infiltration of CD4^−^ T cells [100]. This reaction showed more interferon gamma (IFN-\u03b3) but no IL-4, suggesting a Th1-skewed immune activation [8,101]. By avoiding pro-inflammatory byproducts and allowing localized immunological modulation, the zeolite framework offered a chemically inert matrix for continuous and targeted NO release [8,102,103]. These results suggest Ze–NO as a possible candidate for future dermatologic treatments including psoriasis, wound healing, and UV-induced skin damage [8]. Refer to Figure 3 for a visual representation of ointment application.

#### 3.1.7. Nanozeolite–Starch Thermoplastic Hydrogels and Wound Healing

Dressing wounds has an important role in urgent wound management and treatment, and is able to accelerate wound healing. Siavash et al. (2017) prepared a starch-based nanocomposite hydrogel scaffold, reinforced with zeolite nanoparticles (nZs), and camomile extract, an herbal drug, was added for the acceleration of wound healing (starch/extract/4 wt% nZ) [71]. This dressing was tested on 48 rat animal specimens, as well as five patients with chronic wounds. This study found that, of the four groups of rats, the starch/extract/4 wt% nZ group showed a significant difference in wound size at days 7, 14, and 21 after burn injury given to all the rats, when compared to the other three test groups. Histopathological scoring revealed improvement of epithelization, fibrosis, and angiogenesis scores, with a decrease in inflammation score, in the starch/extract/4 wt% nZ group. In the group of five patients, four had non-healing ulcers after regular care, with the last patient suffering from a rapidly expanding ulcer; after treatment with starch/extract/4 wt% nZ, all patients experienced ulcer improvement and healing, with no reported hypersensitivity reactions or complications.

Based on the results of the animal and pilot study, zeolite use in wound dressings shows strong benefits to wound healing, while shortening healing time, and lessening the severity of the wound, without showing any adverse effects from administration [71,82,94,95]. Figure 3 demonstrates the application of a hydrogel.

#### 3.1.8. Summary of Wound Healing

Zeolite-based materials, particularly ZIF-8, have demonstrated significant potential in wound healing applications. Their multifunctionality, including drug delivery, antimicrobial properties, immune modulation, and oxidative stress reduction, positions them as viable alternatives to traditional wound care therapies. Clinoptilolite, a natural zeolite, has also shown promise in cutaneous wound management. Future research should focus on optimizing formulations, conducting large-scale clinical trials, and evaluating long-term safety to facilitate their clinical translation.

### 3.2. Antimicrobial and Treatment of Infections

Skin infections are highly prevalent, arising from both iatrogenic factors and breaches in skin integrity, which permit the invasion of various microorganisms and disrupt the host’s physiological balance. The persistent challenge of antimicrobial resistance underscores the urgent need for innovative strategies to regain control over infectious processes. In this context, zeolites have emerged as a promising therapeutic modality, particularly for topical applications, offering an effective alternative to systemic antibiotics in the treatment of diverse infections. Zeolites demonstrate antimicrobial potential against common pathogens such as *Staphylococcus aureus* and *Staphylococcus epidermidis*, as well as more resistant strains, including methicillin-resistant *Staphylococcus aureus* (MRSA) and *Pseudomonas aeruginosa* [12,14,72,73,104,105,106].

#### 3.2.1. Metal–Ion Functionalized Zeolites

Various studies have looked into several types of zeolites for treating bacterial specificity, focusing on metal–ion interactions with zinc (Zn^2^⁺), silver (Ag⁺), and copper (Cu^2^⁺) ions with both natural and synthetic clinoptilolite rock [12,51,72,107,108,109,110,111,112]. Zinc is among the most extensively studied ions and has been shown to enhance the bactericidal activity of zeolitic frameworks. Its antimicrobial mechanism is largely attributed to the generation of reactive oxygen species (ROS) through interaction with thiol groups in bacterial respiratory enzymes, resulting in oxidative damage to lipids, proteins, and DNA [60,61,112].

Copper-doped zeolites (nZH-Cu) have also demonstrated significant activity against *Escherichia coli* and *Staphylococcus aureus*, with minimal inhibitory concentrations (MICs) observed at 1 mg/mL [111]. Unlike the undoped control (nZH), these copper-loaded variants show promise for topical applications, particularly in chronic wound care, though further investigations are warranted to optimize formulation and delivery routes.

Milenković et al. (2017) investigated the antibacterial activity of natural and synthetic zeolite A doped with Ag, Cu, and Zn ions, observing antibacterial activity within the first hour of contact with *E. coli* [62]. The study confirmed that the bactericidal effect resulted from the diffusion and activity of the metal ions themselves, thus supporting their use in localized delivery systems.

In parallel, silver–silica nanocomposites such as HeiQ AGS-20 have demonstrated potent antimicrobial action through controlled Ag⁺ ion release, disrupting microbial metabolism and inducing cell death [2,31,32]. Notably, when silver is incorporated into a zeolitic matrix, the resulting composites exhibit enhanced efficacy (up to tenfold) compared to traditional nanocomposites [32]. This is attributed to flame spray pyrolysis synthesis, which reduces nanoparticle agglomeration and ensures targeted ion delivery [32].

Zeolites loaded with Ag⁺, Zn^2^⁺, and Cu^2^⁺ ions have shown broad-spectrum antimicrobial effects against *S. aureus*, *E. coli*, *Bacillus cereus*, and *P. aeruginosa*, with MICs as low as 16 µg/mL [2,3,9,12,14,31]. Antifungal activity have been observed against *Candida albicans*, *C. glabrata*, *Aspergillus niger*, and *Penicillium vinaceum*, particularly for Zn^2^⁺ and Cu^2^⁺ doped variants, at concentrations up to 1024 µg/mL [9,14,15].

These findings confirm the dermatological value of metal-ion functionalized zeolites, especially in wound care and infection prevention settings. Through their controlled release properties, structural stability, and broad-spectrum efficacy, these materials hold strong potential for the localized treatment of skin infections, with reduced risk of systemic toxicity and antimicrobial resistance.

#### 3.2.2. Zeolite X and Other Frameworks

Kwakye-Awuah et al. (2008) synthesized zeolite X at the University of Wolverhampton, UK, and investigated its antibacterial efficacy against three bacterial species: *Staphylococcus aureus*, *Escherichia coli*, and *Pseudomonas aeruginosa* [105]. Their investigations showed that bacterial colonies were completely eradicated after one hour of treatment in a tryptic soy broth (TSB) medium. Notably, the study examined the long-term antibacterial efficacy of silver-loaded zeolite X. The scientists discovered that silver ions were released more quickly in cultures containing *E. coli* than *S. aureus* and *P. aeruginosa*. This differential release implies that silver-zeolite X may be a more effective antimicrobial agent for infection prevention and therapy than traditional antimicrobial agents. Preliminary data would also suggest that further investigation is still needed to avoid levels of toxicity within both animal and human trials.

(crystal composition: Na_80_[Si_112_Al_80_O_384_]·260H_2_O)

Another set of researchers investigated the use of several zeolitic structures, especially zeolite Beta, Rho, and Paulingite, as silver ion carriers [73]. Their findings revealed that the development of silver nanoparticles and clusters inside zeolite X was directly related to increasing silver content (varying from 3 to 6 wt%), which dramatically improved antibacterial activity by reducing the samples’ minimum inhibitory concentration (MIC). Among the examined materials, zeolite Rho had the highest antibacterial activity, whereas zeolite Beta had the lowest. The discrepancies in antibacterial effectiveness were linked to changes in the shapes and distribution of silver inside the distinct zeolite frameworks.

#### 3.2.3. ZSM-5 for Antibiotic and Metal Ion Delivery

ZSM-5 is a synthetic zeolite with an MFI-type framework that can be synthesized using three main methods: organic and inorganic amine templating systems, load-assisted systems, and both hydrothermal and non-hydrothermal pathways [63,64,65,66,67]. Ongoing research has expanded the knowledge and use of this framework, emphasizing its consistent channel dimensions, inherent acidity, and significant shape selectivity afforded by its medium pore size (~6 Å) [68,69]. Recent improvements and material engineering have validated ZSM-5 as a feasible antibiotic delivery platform, notably for infection control and management [70].

The organized microporous architecture, large Brunauer–Emmett–Teller (BET) surface area, and surface functional groups all contribute to the drug’s increased loading capacity and sustained release qualities. Gentamicin-loaded ZSM-5, in particular, exhibited extended antimicrobial release, greatly lowering bacterial adherence and suppressing *Staphylococcus epidermidis* biofilm formation. The favourable biocompatibility, efficient delivery capabilities, and potent bactericidal activities of ZSM-5 zeolites highlight their promising potential in dermatological and wound care applications [70]. There are currently no studies evaluating gentamicin-loaded ZSM-5 for skin infections; however, as well as facilitating tissue regeneration [113], particularly for infections of the mesenchymal stem cell lineage, earlier evidence suggests a possible role for employing zeolite A with chitosan [114]. It should be highlighted that with more research and innovation into particular medicines for treating skin infections, the ZSM-5 framework might minimize the usage of systemic antibiotics.

Sánchez et al. demonstrated that silver-functionalized ZSM-5 zeolite exhibits potent antimicrobial activity [115]. The incorporation of Ag⁺ ions significantly inhibited the growth of key pathogens, including *Escherichia coli*, *Pseudomonas aeruginosa*, and *Candida albicans*—organisms of major clinical concern. Clear zones of inhibition and kinetic data confirmed sustained microbial suppression. These findings highlight Ag⁺-modified ZSM-5 as a promising candidate for antimicrobial applications, particularly in dermatological and wound care settings where infection control is critical [115].

#### 3.2.4. Zeolites for Candida Auris and Other Fungal Infections

A clinical hospital in Saudi Arabia cultured 19 strains of *Candida auris* (C. auris) from patient samples—an emerging, multidrug-resistant fungal pathogen known for its persistence in hospital environments and its ability to form resilient biofilms. These isolates were characterized by high levels of antifungal resistance, particularly to azoles such as fluconazole, due to mutations in key genes including *ERG11* and *TAC1B* [83,116,117,118,119]. In response to the growing threat posed by *C. auris*, this study investigated the antifungal potential of nanomaterials—specifically Ag-silicalite-1 and 4 wt% Ag/TiZSM-5 zeolites—as therapeutic interventions targeting biofilm-forming, drug-resistant strains [116]. As the study suggested, further investigation is warranted, especially in high-dependency units within hospitals.

Torres et al. (2019) investigated the therapeutic use of Detoxsan^®^, a zeolite-based paste containing clinoptilolite and mordenite, for treating superficial fungal infections and intertrigo in a tropical clinical setting [15]. The paste utilizes the zeolite’s high binding capacity for histamine and moisture, creating a dry, non-irritant barrier that facilitates skin recovery without pharmacological agents [84,120,121]. Due in large part to the anti-inflammatory and antimicrobial properties of zeolite, patients who received semi-daily treatment demonstrated complete skin restoration in 10–15 days without experiencing any adverse effects [15,121,122]. The mineral structure of zeolite outperformed swelling clays like montmorillonite in creating a stable, non-aqueous therapeutic layer [123,124,125], and the addition of petrolatum and squalane promoted skin adherence and moisture balance [15,52,53,54].

#### 3.2.5. ZIF-8 for Zinc Interference Therapy

Recent research into zinc ion interference therapy highlights the antimicrobial potential of zeolitic frameworks, particularly ZIF-8, a zinc-based metal–organic framework (MOF), for targeting multidrug-resistant (MDR) bacteria and fungi. ZIF-8’s acid-responsive porous structure enables the pH-triggered release of Zn^2^⁺, leading to oxidative stress, protein dysfunction, and DNA damage in pathogens such as *Staphylococcus aureus*, *Escherichia coli*, and *P. acnes* [55,56,57,112].

Functionalized ZIF-8 composites—including ICG@ZIF-8/PDA/Ag and TZH (tetracycline-loaded ZIF-8 with hyaluronic acid)—demonstrated superior bactericidal effects by combining Zn^2^⁺ overloading with photothermal or antibiotic synergy, resulting in >99% bacterial inhibition [55,58,112]. In fungal applications, ZIF-8’s Zn^2^⁺ release disrupted fungal cell walls, while coatings such as polydopamine (PDA) improved biocompatibility and bacterial targeting [59,74,112].

Moreover, ZnO-based nanomaterials, although effective against *Candida albicans*, present higher cytotoxicity risks compared to ZIF-8. As a result, ZIF-8 is favoured for its controllable release kinetics, lower mammalian toxicity, and enhanced targeting capability, especially when modified with polymers or bioligands [55,75,76,112].

These findings position ZIF-8 and its derivatives as promising agents for clinical intervention in multidrug-resistant (MDR) bacterial and fungal infections, with further development needed to enhance targeting precision and in vivo safety.

#### 3.2.6. Silver Sulfadiazine–Zeolite Composites for Burn-Related Infections

Yassue-Cordeiro et al. (2019) developed a chitosan-based wound dressing incorporating silver sulfadiazine (AgSD)-loaded NaY zeolite to improve infection control in burn patients [77]. The NaY zeolite, characterized by its high surface area and porous structure (Si/Al ≈ 2.5), was used to enable a controlled, sustained release of AgSD, addressing the limitations of conventional creams that require frequent replacement [77,78,108,126,127,128].

The composite films showed potent antimicrobial activity against *Staphylococcus aureus*, *Escherichia coli*, *Pseudomonas aeruginosa*, and *Candida albicans*—key pathogens in burn-related infections [129,130]. Compared to standard formulations, the AgSD–zeolite films provided prolonged silver ion release, enhancing bactericidal efficacy while minimizing cytotoxic peaks (Table 2 and Table 3) [85,131].

**Table 3:** Zeolite Applications Categorized by Dermatological Condition.

Condition/Use Case	Relevant Zeolites	Key Outcomes	Study Type	References
Chronic wounds/Diabetic ulcers	ZIF-8, Clinoptilolite, CaCu-Zeolite, NO-Zeolite, Nanozeolite Hydrogels	Enhanced healing, reduced inflammation, infection control	In vitro, in vivo, pilot clinical	[12,14,22,25,43,44,45,46,47,48,49,50,52,53,54,55,56,57,58,59]
Burn wound infections	NaY Zeolite + AgSD, ZIF-8	Prolonged silver release, reduced bacterial load, stable formulation	In vitro, formulation studies	[51,73,74,75,76,77,78]
Acne vulgaris	Zn-Clinoptilolite, ZSM-5, ZIF-8 Microneedles	Reduced *P. acnes*, inflammation, enhanced drug delivery	In vitro, in vivo, clinical	[14,54,67,89,90,91,92,102]
Skin detoxification/Pollution exposure	Clinoptilolite, Modified Zeolites (3%)	Heavy metal removal, antioxidant protection	Clinical, formulation tests	[7,17,22,25,49]
Fungal infections/Intertrigo	Detoxsan^®^, Ag-Zeolites, TiZSM-5	Rapid symptom resolution, safe barrier protection	Clinical observational	[15,83,84]
Skin brightening/Antiageing	Gold-Zeolite Nanocomposites, ZIF-8 (with actives)	Wrinkle and melanin reduction, improved skin texture	Human clinical	[13,85,86]
Peri-implantitis/Oral ulcers	ZIF-8 Microneedles, HMME@ZIF-8 Hydrogel	Targeted drug delivery, bacterial inhibition, tissue regeneration	Animal, in vitro	[51,52,53,54,55,56,57,58,59,89]
Body odour/Deodorant alternative	Ag-Zeolite	24 h protection, safer than triclosan	In vitro, clinical comparison	[2,3,9,31,32]
Skin cancer/Immunotherapy	ZIF-8, Clinoptilolite, MIT@ZIF-8	Immune activation, tumour regression, reduced toxicity	In vivo (murine), mechanistic studies	[8,49,52,132,133]

Physicochemical analyses (XRD, FTIR, SEM) confirmed the successful integration of AgSD into the zeolite matrix, and thermal studies demonstrated improved film stability and moisture retention, both critical for burn wound environments [56,86,130]. Although some cytotoxicity was observed at higher concentrations, the dressing was considered safe in a dose-dependent manner (aligned with ISO 10993-09 regulations) [77,134].

The inclusion of silver sulfadiazine into NaY zeolite inside chitosan films provides a potential method for controlling infections in burn patients, with controlled antibacterial release and broad-spectrum activity. This method solves important disadvantages of standard AgSD creams, such as short duration of action and cytotoxicity (Table 3).

## 4. Zeolites in Cosmetology and Dermatological Conditions

### 4.1. Cosmetology

Zeolites have garnered increasing attention in dermatology and cosmetology due to their physicochemical versatility, high biocompatibility, and structural adaptability. These microporous materials possess high surface area, cation-exchange capabilities, and chemical resilience, enabling their use as drug delivery platforms, stabilizers, and active participants in skin therapy [3,4,5,13,16,17,34,87,88,89,90,91,92,93,135,136,137,138]. Unlike traditional excipients, zeolites play multifunctional roles—extending beyond passive delivery to include antioxidant, detoxifying, and anti-inflammatory actions.

In cosmetic formulations, zeolites are now incorporated into creams, masks, microneedle patches, and transdermal systems, where they address complex therapeutic targets such as pigmentation, oxidative stress, ageing, and environmental damage. Their compatibility with both hydrophilic and lipophilic compounds, along with structural tunability, has made them central to the development of controlled-release systems that balance efficacy with tolerability in a variety of skin types.

#### 4.1.1. Zeolite Nanocomposites for Antiageing and Brightening Applications

Recent advancements have led to the development of zeolite-based nanocomposites decorated with gold nanoparticles, exemplifying the integration of structural delivery control and therapeutic enhancement. A pivotal study by Lee et al. (2023) evaluated formulations incorporating sulforaphane, adenosine, and niacinamide into gold-decorated zeolite carriers (Au-ZL NCs), revealing dramatic improvements in clinical outcomes [13]. In a four-week human trial, these systems achieved a 203% improvement in wrinkle depth and a melanin reduction of up to 222% compared to standard actives alone [13]. This enhancement was attributed to increased dermal penetration, molecular stability, and the synergistic antioxidant effects of gold (Table 2 and Table 3).

Supporting these findings, foundational work by Pestryakov et al. (2005) [135] demonstrated that zeolite frameworks provide a stable microenvironment for gold nanoparticle formation and dispersion. By controlling particle agglomeration, zeolites preserved catalytic activity and surface area, factors essential for sustained topical action. Further, González et al. (2021) [87] engineered zeolite nanostructures functionalized with amine groups to support ultra-small gold nanocluster adhesion. This platform facilitated protein interactions beneficial for wound healing and reduced inflammatory cytokine production, pointing to potential use in post-treatment skincare following microneedling or laser therapy.

Together, these studies underscore the value of zeolite–gold nanocomposites as multifunctional cosmeceutical agents that enhance the effectiveness, stability, and bioactivity of cosmetic actives.

#### 4.1.2. Zeolitic Delivery Systems for Pigmentation Disorders and Inflammatory Skin Conditions

Beyond their mechanical and stabilizing properties, zeolites have been explored for improving the dermal absorption of challenging hydrophilic compounds. A notable example involves the encapsulation of tranexamic acid (TA) within zeolitic imidazolate frameworks (ZIF-8), yielding a TA@ZIF-8 delivery system designed to treat melasma and rosacea [88]. Tang et al. (2024) reported that this system facilitated the expression of aquaporin-3 (AQP-3) in keratinocytes, enhancing transepidermal water and solute transport [88]. In vitro analyses revealed reduced melanin synthesis and suppressed inflammatory cytokines, while in vivo results demonstrated decreased erythema and visible skin brightening without adverse effects. The ability to deliver TA topically—without systemic exposure or associated risks—marks a significant advancement for pigment and inflammation-targeted formulations.

#### 4.1.3. Zeolite Microneedles: From Concept to Clinical Readiness

Zeolite microneedles (Z-MNs) represent a sophisticated evolution in transdermal drug delivery systems. Their design leverages zeolites’ mechanical integrity, porosity, and bioinertness to achieve minimally invasive, long-acting dermal administration of both small molecules and macromolecules.

The concept was initially proposed by Ramović et al. (2019) [89], who envisioned the use of zeolite microneedles for delivering large bioactive compounds such as collagen. Their hypothesis was based on zeolites’ superior mechanical strength and ability to house sizable molecules within their framework. Experimental validation soon followed. Wong et al. (2007) fabricated sintered aluminosilicate microneedles and confirmed their ability to penetrate porcine skin, maintain structural integrity, and facilitate sustained transdermal delivery of model compounds [90].

Subsequent research further examined the mechanical resilience of Z-MNs under various environmental conditions, revealing their superiority over dissolvable polymers in terms of shape retention, osmotic stability, and long-term performance. Importantly, Poon’s work also emphasized the feasibility of integrating actives during the fabrication process, allowing the microneedles to serve simultaneously as delivery devices and stabilizing matrices [91].

**Figure 5:**
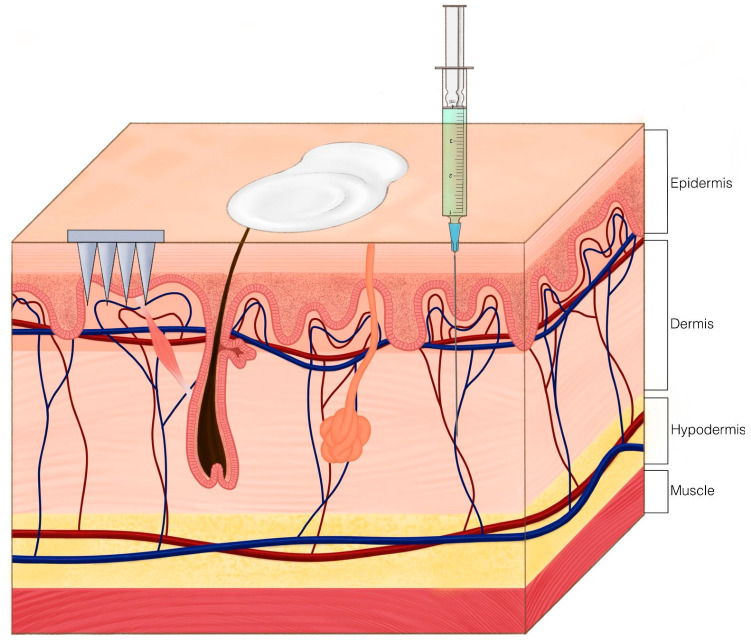
Cross-sectional comparison of transdermal delivery methods: microneedles (**left**), topical application (**centre**), and intradermal injection (**right**). Microneedles penetrate the stratum corneum to deliver active agents into the viable epidermis and dermis, offering a targeted, minimally invasive alternative to conventional approaches. In cosmetology, zeolite-based microneedles—such as ZIF-8 and Zn–clinoptilolite systems—enable sustained delivery of agents like tranexamic acid, sulforaphane, and erythromycin, enhancing efficacy while reducing irritation and systemic exposure [13,87,88,90,137].

Two follow-up studies explored the pharmacokinetics of macromolecular delivery using silica-based Z-MNs. By tuning the inter- and intracrystalline pore size, the team achieved controlled release of compounds ranging from 20 to 100 kDa. These findings were validated in vivo, where insulin-loaded Z-MNs maintained therapeutic plasma levels and caused no skin irritation in diabetic rat models (Figure 5) [92,136].

The same group of researchers focused on refining the structural geometry of Z-MNs, correlating tip sharpness, porosity, and taper angle with skin penetration efficiency and patient comfort. These design variables were found to significantly influence drug absorption kinetics, supporting the tailoring of Z-MNs for specific dermatological applications [93].

In a comprehensive review, Kulkarni et al. (2022) affirmed the relevance of Z-MNs in cosmeceutical design, highlighting their compatibility with both hydrophilic and lipophilic agents, their ability to prevent enzymatic degradation, and their adaptability to home-use delivery systems [137]. The convergence of structural engineering and cosmeceutical science in Z-MNs positions them as intelligent, next-generation devices for long-acting skincare. Recent progress in micromolding and solvent-casting methods already used for other types of microneedles, which shows that Z-MNs can likely be produced at scale and at relatively low cost [139]. Zeolites themselves are inexpensive, and their properties are compatible with automated manufacturing, making Z-MNs a practical option for wider clinical and commercial use. In our exploration of microneedle technologies, we found that conventional fabrication methods such as laser ablation, photolithography, and three-dimensional printing often result in limited biocompatibility, which makes them less suitable for some wound environments [140].

The microneedle types shown in Figure 4—solid, coated, and hollow—demonstrate how zeolites can be used in different delivery formats. This structural flexibility helps improve drug targeting and release, while also supporting designs that are easier to manufacture and adapt for specific dermatological treatments. It is also important to acknowledge that production costs may vary depending on the choice of materials and the location of manufacturing.

#### 4.1.4. Detoxification and Environmental Protection: Zeolites as Skin Guardians

Zeolites also act as environmental protectants, with notable applications in urban skincare. Their ion-exchange properties allow for the adsorption of toxic metals such as cadmium and nickel, commonly found in polluted environments. Pesando et al. (2022) [3] conducted a clinical trial comparing 1% and 3% zeolite-containing creams. The 3% formulation significantly outperformed both lower concentrations and placebo in removing heavy metals while preserving skin hydration and barrier function. These findings suggest that zeolites serve as both chemical detoxifiers and physical skin protectants, reinforcing their role in antipollution skincare.

Further, Pandya et al. (2024) [4] emphasized zeolites’ stabilizing effects on volatile and oxidation-prone ingredients such as essential oils and antioxidants. Their ability to prolong shelf life and improve active delivery supports their inclusion in moisturizers, serums, and detoxifying formulations.

#### 4.1.5. Functionalized Zeolite Hybrids and Controlled Release Systems

Innovative zeolite-based hybrids further extend their application in skincare through surface modifications with bioactive polymers. Wawrzyńczak et al. (2023) [138] synthesized hierarchical zeolites functionalized with hyaluronic acid, heparin, and inulin, combining biocompatibility with structural porosity. These hybrids demonstrated pH-responsive release profiles, improved water retention, and anti-inflammatory properties, making them ideal for sensitive or post-procedural skin.

Complementary work by Rimoli et al. (2008) [5] confirmed that drug release from zeolites can be precisely controlled through structural tuning. Their studies with ketoprofen and theophylline revealed that zeolites with high Si/Al ratios permitted slow, sustained release while minimizing initial burst effects. This behaviour is especially beneficial in cosmeceuticals requiring prolonged action and reduced irritation, such as overnight repair creams or wrinkle-smoothing gels.

### 4.2. Antiacne

Acne vulgaris remains a prevalent dermatological disorder, affecting individuals of all ages and often requiring long-term therapeutic management. Conventional treatments such as benzoyl peroxide, antibiotics, and retinoids present challenges including antimicrobial resistance, skin irritation, and reduced patient adherence [141]. In recent years, Zn-exchanged clinoptilolite zeolite has emerged as a promising biomaterial for acne therapy, owing to its antibacterial, anti-inflammatory, and controlled drug release capabilities [14]. This section explores the role of Zn-exchanged clinoptilolite as an antibiotic carrier, its targeted antiacne applications, and its broader antimicrobial potential [12,14,142], with an emphasis on clinical trials and case studies.

#### 4.2.1. Zn-Exchanged Clinoptilolite as an Antibiotic Carrier

Zn-exchanged clinoptilolite zeolite has gained significant attention in dermatology and antimicrobial therapy due to its ion exchange properties, controlled drug release capacity, and broad-spectrum antimicrobial effects [14,142]. In particular, Zn^2^⁺–clinoptilolite formulations have been tested for acne treatment and wound healing [12], showing efficacy in reducing *Propionibacterium acnes (P. acnes)* populations, modulating inflammation, and enhancing topical drug delivery (Table 3) [14,142]. This section reviews the latest clinical studies assessing the antimicrobial and antiacne properties of Zn-exchanged clinoptilolite zeolite, highlighting its therapeutic potential and clinical efficacy.

#### 4.2.2. Mechanism of Action

Clinoptilolite, a naturally occurring zeolite, is widely recognized for its cation exchange properties, allowing it to adsorb and release metal ions in a controlled manner [72,143]. The incorporation of zinc ions (Zn^2^⁺) within the zeolite structure enhances its antimicrobial activity, as Zn^2^⁺ exhibits bacteriostatic and bactericidal effects by:

- Disrupting bacterial cell membranes and impairing intracellular enzymatic functions [144,145].
- Reducing the proliferation of *P. acnes*, a key acne-causing pathogen [146].
- Mitigating inflammation through inhibition of pro-inflammatory cytokines [12].

#### 4.2.3. Controlled Drug Release in Dermatological Applications

The potential of Zn-exchanged clinoptilolite as a controlled drug release method has been thoroughly explored in dermatological applications. Notably, investigations have shown that Zn–clinoptilolite greatly extends the release of erythromycin, a commonly used antibiotic for acne. This prolonged-release method improves therapeutic efficacy while simultaneously reducing systemic exposure and related side effects, making it a more acceptable therapy choice for patients [12,147,148,149]. This administration method enhances patient adherence by lowering application frequency, resulting in optimal long-term therapy outcomes.

A unique composite formulation containing zeolite, zinc, and erythromycin has been created particularly for acne treatment. This novel strategy capitalizes on zinc’s capacity to decrease inflammation and sebum production, while erythromycin directly targets acne-causing bacteria. This formulation’s efficacy has been recognized through patent filings, highlighting its potential as an enhanced treatment solution [14,72]. Importantly, tests show that erythromycin’s antibacterial potency stays unchanged when incorporated into the zeolite matrix, implying that Zn–clinoptilolite may improve the stability and efficiency of traditional antibiotics.

Furthermore, the microporous structure of zeolites plays a crucial role in enabling controlled and sustained drug release, thereby minimizing localized side effects. A subsequent study investigated the use of ZSM-5 zeolite as a gentamicin delivery system and found that this delayed-release mechanism successfully decreases irritation in skin infections by guaranteeing a progressive and consistent drug release profile [70]. These findings underscore zeolites’ therapeutic adaptability, establishing Zn-clinoptilolite as a prospective contender for next-generation acne therapies and dermatological drug delivery platforms.

#### 4.2.4. Zinc in Acne Management: A Safer, Effective Alternative to Antibiotics

Clinical trials were conducted to assess the efficacy of treating acne vulgaris of different grades and severity, while zinc acetate has demonstrated therapeutic value as an adjunct in the topical treatment of acne, particularly in formulations combined with 4% *w*/*v* erythromycin. In vitro findings show that erythromycin-resistant *Propionibacterium acnes* strains remain susceptible to zinc acetate, supporting its inclusion to potentially enhance antibacterial efficacy [150]. In clinical settings, both erythromycin alone and erythromycin–zinc combinations significantly reduced total *P. acnes* counts and erythromycin-resistant populations (*p* < 0.01). The zinc–erythromycin combination significantly reduced acne severity in both inflamed and non-inflamed lesions (*p* < 0.001). Notably, most patients with high levels of resistant bacteria improved clinically by more than 50%, even when treated with the zinc-containing formulation.

A meta-analysis and systematic review revealed that 2445 acne vulgaris patients across 25 different studies were found to have significantly low serum zinc levels compared to control subjects [151]. Subjects who were treated with zinc had a noteworthy improvement in mean papule count compared to those who were not treated with zinc. There was no significant difference in the occurrence of gastrointestinal side effects, such as nausea, vomiting, and abdominal pain [152,153,154,155,156], between zinc supplementation and comparison groups. Other side effects from topical uses of zinc noted dryness and acne flares.

An open-label study compared oral zinc sulphate to lymecycline, a common acne therapeutic, examined 100 patients with mild to moderate papulopustular acne [157]. The authors administered a daily dose of 400 mg of zinc sulphate for 12 weeks (as guided by the literature) [158,159], and the results showed no statistical difference between zinc and lymecycline. However, the results from the acne quality of life questionnaire (AQoL) increased at both 4 and 12 weeks, with significantly higher values recorded in the group receiving zinc sulphate therapy.

In summary, zinc—whether used topically in combination with erythromycin, lymecycline, minocycline [157] or administered orally—offers a clinically effective and well-tolerated alternative to antibiotics, helping to manage acne while addressing the critical issue of rising antimicrobial resistance, though a larger scale of candidates would need to be evaluated and compared to all grades of acne vulgaris.

#### 4.2.5. Other Modalities for Treating Acne: Photodynamic Therapy

Photodynamic therapy (PDT) eliminates targeted cells through the use of a light-sensitive compound known as a photosensitizer, which becomes activated when exposed to a specific light source, such as lasers or LEDs. PDT is commonly applied as a localized treatment, focusing on specific areas of the body [160]. This dermatological advancement plays a significant role in treating acne as well as various forms of skin cancer. Moy, Frost, and Moy (2020) reported reductions in cytokine activity and bacterial presence, leading to decreased inflammation following multiple treatment sessions [160,161].

Topically applied photosensitizers can penetrate into the deeper dermal layers, contributing to improvements in certain types of acne scarring and stimulating collagen remodelling. In cases of active acne, PDT effectively targets the sebaceous glands, heating and ablating their activity. Furthermore, PDT exerts a potent antibacterial effect by disrupting the *Propionibacterium acnes* biofilm [162,163], thereby enhancing its therapeutic efficacy. Aminolevulinate (ALA) is the common agent used for acne and some skin cancers, but the authors mentioned 4% for acne and acne scarring [160].

On meta-analysis measured 13 RCTs involving 701 participants showed some PDT groups may improve clinical effectiveness for inflammatory acne when compared to the visible PDT or medication treatment group. The findings align with previous review research [164,165], which suggested that PDT may outperform placebo, non-treatment, topical therapy, and other comparable treatments for inflammatory acne. What is also mentioned is the type of photosensitizer used, ALA-PDT, Methyl-aminolevulinate (MAL-PDT) [132,166], and liposomal methylene blue (LMB-PDT) [133,167] effectively improved the clinical efficacy for inflammatory acne [133,164,168]. The authors expressed the need to review the drug efficacy of each agent in response to treating acne.

#### 4.2.6. Promising Future for Zeolitic Integration with PDT for Treating Acne Vulgaris

Emerging acne therapies are increasingly incorporating advanced materials and targeted delivery systems to enhance treatment efficacy. A promising multi-modal approach involves the integration of zeolites, particularly zinc-exchanged clinoptilolite [169] and zeolitic imidazolate framework-8 (ZIF-8), with microneedling and photodynamic therapy (PDT) [57]. This combination improves drug penetration, boosts antimicrobial activity, and mitigates inflammation—marking a significant advancement toward non-antibiotic, precision-based acne treatments.

A recent study by Wen et al., 2021 [57], highlights the development of ZIF-8-ICG@MNs, a microneedle patch loaded with a bioresponsive nanoplatform for treating acne vulgaris. In this system, ZIF-8 plays a critical role by stabilizing the photosensitizer indocyanine green (ICG), enabling efficient generation of reactive oxygen species (ROS) upon near-infrared (NIR) irradiation. Furthermore, ZIF-8 selectively degrades in the acidic microenvironment of acne lesions, releasing antimicrobial Zn^2^⁺ that disrupts bacterial membranes and enhances therapeutic outcomes [57,145]. This synergistic chemo-photodynamic therapy demonstrated significant antiacne effects both in vitro and in vivo, underscoring the potential of ZIF-8 for enhanced drug delivery, targeted degradation, and robust antimicrobial performance. Further investigation is warranted to evaluate the efficacy of this synergistic therapy across different grades of acne severity and against a broader spectrum of acne-associated bacteria beyond *P. acnes*.

### 4.3. Integration, Customization, and Future Perspectives

Zeolites’ structural diversity and functional plasticity allow them to serve as platforms for diverse dermatological applications. Servatan et al. (2020) emphasized the integration potential of zeolites into broader drug delivery systems, particularly through surface modification techniques that enable targeted release and enhanced biocompatibility [16]. Their compatibility with microneedles, nanoparticles, and cream matrices underscores their adaptability in both medical and cosmetic dermatology.

As formulation science moves toward multi-targeted, patient-personalized skincare, zeolites offer a rare combination of robustness, customizability, and biological safety. Ongoing innovations in surface engineering, hybrid composites, and transdermal technology suggest that zeolites will continue to occupy a central role in next-generation cosmeceuticals—advancing from passive carriers to active agents in skin restoration, protection, and rejuvenation.

The potential of zeolite integration in dermatology rests in its ability to promote multimodal, patient-centred therapy through structural customization and intelligent formulation design. As evidenced by emerging antiacne applications, zinc-exchanged zeolites and frameworks such as ZIF-8 are pushing the boundaries of traditional treatment by combining antimicrobial efficacy, controlled release, and compatibility with adjunctive techniques such as photodynamic therapy and microneedling [14,57,142,169]. These advancements highlight a paradigm change from single-function excipients to dynamic delivery systems that offer personalized, long-acting, and resistance-reducing therapies in both cosmetic and therapeutic skin care. Zeolites are therefore positioned not just as adaptable transporters, but also as key agents in the next wave of dermatological innovation.

## 5. Skin Cancer and Zeolites

### 5.1. Zeolites in Chemo-Immunotherapy and Skin Cancer Treatment

Zeolites have emerged as versatile agents in oncologic dermatology, particularly in the realms of chemo-immunotherapy and skin cancer management. Their unique physicochemical properties—including tuneable porosity, ion exchange capacity, and biocompatibility—make them valuable platforms for drug delivery and immunomodulation in cancer treatment.

Recent studies have highlighted the role of zeolitic imidazolate frameworks (ZIF-8) in enhancing immunogenic responses to tumours. ZIF-8 nanoparticles have demonstrated the ability to induce pyroptosis, a form of pro-inflammatory programmed cell death, via the caspase-1/gasdermin D (GSDMD) pathway [170,171]. When ZIF-8 is used to encapsulate mitochondria-depolarizing agents, the pyroptotic response is further intensified, leading to significant suppression of tumour growth in murine 4T1 tumour models [170,171]. Notably, mitoxantrone (MIT)—a type II topoisomerase inhibitor—has been successfully incorporated into ZIF-8 frameworks through a one-pot aqueous-phase synthesis, forming MIT@ZIF-8 complexes. These structures enhance the tumour-specific uptake of MIT while reducing its immunotoxicity in tumour-draining lymph nodes [170].

The pyroptosis-inducing capacity of ZIF-8 also synergizes with MIT’s ability to trigger immunogenic cell death (ICD), a process characterized by the release of damage-associated molecular patterns (DAMPs). This combination facilitates a robust antitumour immune response [170]. In vivo, MIT@ZIF-8 has shown efficacy in both immunologically “hot” colon cancer and “cold” prostate cancer models. Interestingly, in the latter, this treatment reprogrammed the tumour microenvironment, rendering the tumour responsive to anti-CTLA-4 immunotherapy, a significant finding for immunoresistant cancers [170].

Despite these promising outcomes, further studies are required to delineate the full spectrum of therapeutic applications of zeolites in cancer immunotherapy and to evaluate long-term safety and efficacy across tumour types.

### 5.2. Zeolites in Cutaneous Oncology and Skin Cancer Management

Skin cancer remains one of the most frequently diagnosed conditions in dermatology, with increasing global incidence attributed to environmental and lifestyle factors [172]. The early detection and accurate differentiation of benign versus malignant lesions are crucial for optimal management and improved survival outcomes [172,173,174]. Current treatment options for skin cancers, including melanoma, basal-cell carcinoma, and squamous-cell carcinoma, encompass a broad range of strategies—ranging from topical chemotherapeutics and injectable agents to systemic chemotherapy, immunotherapy, radiation, and surgical approaches such as cryosurgery [172,175,176]. Nevertheless, the search for innovative, biocompatible, and targeted therapies continues, and zeolite-based systems have shown emerging promise in this domain.

Zeolites possess several mechanisms of antitumour activity. As discussed, ZIF-8-based systems can induce pyroptosis, thereby suppressing tumour progression through pro-inflammatory immune activation [170,171]. Beyond this, other zeolitic structures have demonstrated the capacity to absorb and release bioactive therapeutic proteins under physiological conditions. Khojewa et al. (2019) examined three zeolite variants—clinoptilolite, chabazite, and natrolite—for their ability to adsorb the antitumour ribonuclease binase and assessed the cytotoxicity of the resulting complexes [177]. All three zeolites achieved approximately 80% protein loading efficiency. Notably, the chabazite–binase complex exhibited enhanced specificity toward Caco-2 colon carcinoma cells, reduced off-target toxicity, and demonstrated time-dependent protein release, highlighting chabazite’s potential as an effective therapeutic protein carrier [177].

In melanoma therapy, clinoptilolite has been studied as a cytotoxic and antimetastatic agent. Pavelic et al. (2001) evaluated a micronized zeolite (MZ) form of clinoptilolite in vitro and in vivo [50]. The compound was shown to downregulate protein kinase B (c-Akt) and upregulate tumour suppressor proteins p21^WAF1/CIP1 and p27^KIP1, thereby arresting cell proliferation. In melanoma-bearing mice treated with oral doses of MZ, tumour growth was significantly suppressed, and survival was extended compared to control animals. A daily dose of 150–200 mg was well tolerated, with no adverse toxicological effects reported [50].

Building upon these findings, further investigation into MZ’s antimetastatic activity revealed immunomodulatory effects contributing to its anticancer profile. In mice inoculated with melanoma cells, MZ administration increased serum levels of lipid-bound sialic acid (LSA), reduced hepatic lipid peroxidation, and promoted heightened graft-versus-host (GVH) responses in lymph node-derived lymphocytes [178]. Peritoneal macrophages exhibited increased superoxide anion production, while nitric oxide (NO) synthesis was completely inhibited. Simultaneously, activation of the NF-κB pathway was observed via p65 subunit nuclear translocation in splenic cells [178]. These immune and biochemical modulations resulted in a statistically significant reduction in melanoma metastases among MZ-treated mice.

Together, these studies underscore the emerging role of zeolites—not only as delivery vehicles but also as active modulators of immune responses and tumour biology—in the management of skin cancers. Their ability to synergize with immunotherapies, enhance localized drug release, and modulate tumour microenvironments positions zeolites as promising candidates in next-generation dermatologic oncology. Continued exploration into zeolite biocompatibility, formulation strategies, and combinatorial regimens will be essential to fully harness their clinical potential in cutaneous malignancies.

## 6. Mechanistic Insight and Future Potential

Zeolites’ diverse properties, particularly those of frameworks like clinoptilolite, ZSM-5, ZIF-8, and variants that have been modified with silver or zinc, make them potentially revolutionary in dermatological treatments. Long-standing difficulties in treating inflammatory dermatoses, chronic wounds, and skin infections are addressed by their capacity to provide controlled, prolonged antimicrobial and anti-inflammatory activity while reducing systemic toxicity. Furthermore, their versatility in contemporary transdermal and topical systems is demonstrated by their incorporation into hydrogels, nanocomposites, and microneedles. The clinical application of zeolite-based treatments is still in its early stages, despite promising in vitro and in vivo results. To completely validate their therapeutic efficacy in dermatology, future research must place a high priority on rigorous clinical trials, formulation standardization, and long-term safety profiling.

Notwithstanding its potential, some obstacles must be overcome prior to the widespread use of zeolite-based treatments in clinical dermatology. Regulatory pathways for novel materials, particularly those involving nanoparticulate or ion-releasing agents, require extensive toxicological and pharmacokinetic validation. Challenges in formulation, such as maintaining batch-to-batch uniformity, assuring biocompatibility, and achieving stability under practical storage and application settings, persist as key issues. Moreover, extensive long-term safety studies in human populations are still absent. Cooperative endeavours among material scientists, physicians, and regulatory agencies will be crucial for advancing the clinical translation and future commercialisation of zeolite-based dermatological therapies.

## Abbreviations

The following abbreviations are used in this manuscript:

MZ	Micronized Zeolite
NO	Nitric Oxide
NF-κB	Nuclear Factor Kappa-Light-Chain-Enhancer of Activated B cells
GVH	Graft-versus-Host
LSA	Lipid-Bound Sialic Acid
PDT	Photodynamic Therapy
ALA	Aminolevulinic Acid
MAL	Methyl Aminolevulinate
LMB	Liposomal Methylene Blue
ZIF-8	Zeolitic Imidazolate Framework-8
ICG	Indocyanine Green
ROS	Reactive Oxygen Species
SEF	Simulated Exudate Fluid
AgSD	Silver Sulfadiazine
BET	Brunauer–Emmett–Teller (surface area analysis)
ZSM-5	Zeolite Socony Mobil–5 (a type of MFI-structured zeolite)
CaCu-ZG	Calcium–Copper Zeolite Gauze
MOF	Metal–Organic Framework
MIC	Minimum Inhibitory Concentration
